# Quality of Midwife-provided Intrapartum Care in Amhara Regional State, Ethiopia

**DOI:** 10.1186/s12884-017-1441-2

**Published:** 2017-08-16

**Authors:** Tegbar Yigzaw, Fantu Abebe, Lalem Belay, Yewulsew Assaye, Equlinet Misganaw, Ashebir Kidane, Desalegn Ademie, Jos van Roosmalen, Jelle Stekelenburg, Young-Mi Kim

**Affiliations:** 1 Jhpiego, Addis Ababa, Ethiopia; 2 Jhpiego, Bahir Dar, Ethiopia; 3 Faculty of Earth and Life Sciences, Vrije Universiteit, Amsterdam, Netherlands; 4 Department of Obstetrics and Gynecology, Leeuwarden Medical Centre, Leeuwarden, Netherlands; 5 Department of Health Sciences, Global Health, University Medical Centre Groningen, University of Groningen, Groningen, Netherlands; 6 Jhpiego, Baltimore, USA

**Keywords:** Labor, childbirth and immediate postpartum care, Competence, Enabling environment, Physical resources, Performance and quality improvement

## Abstract

**Background:**

Despite much progress recently, Ethiopia remains one of the largest contributors to the global burden of maternal and newborn deaths and stillbirths. Ethiopia’s plan to meet the sustainable development goals for maternal and child health includes unprecedented emphasis on improving quality of care. The purpose of this study was to assess the quality of midwifery care during labor, delivery and immediate postpartum period.

**Methods:**

A cross-sectional study using multiple data collection methods and a 2-stage cluster sampling technique was conducted from January 25 to February 14, 2015 in government health facilities of the Amhara National Regional State of Ethiopia. Direct observation of performance was used to determine competence of midwives in providing care during labor, delivery, and the first 6 h after childbirth. Inventory of drugs, medical equipment, supplies, and infrastructure was conducted to identify availability of resources in health facilities. Structured interview was done to assess availability of resources and performance improvement opportunities. Data analysis involved calculating percentages, means and chi-square tests.

**Results:**

A total of 150 midwives and 56 health facilities were included in the study. The performance assessment showed 16.5% of midwives were incompetent, 72.4% were competent, and 11.1% were outstanding in providing routine intrapartum care. Forty five midwives were observed while managing 54 obstetric and newborn complications and 41 (91%) of them were rated competent. Inventory of resources found that the proportion of facilities with more than 75% of the items in each category was 32.6% for drugs, 73.1% for equipment, 65.4% for supplies, 47.9% for infection prevention materials, and 43.6% for records and forms. Opportunities for performance improvement were inadequate, with 31.3% reporting emergency obstetric and newborn care training, and 44.7% quarterly or more frequent supportive supervision. Health centers fared worse in provider competence, physical resources, and quality improvement practices except for supportive supervision visits and in-service training.

**Conclusions:**

Although our findings indicate most midwives are competent in giving routine and emergency intrapartum care, the major gaps in the enabling environment and the significant proportion of midwives with unsatisfactory performance suggest that the conditions for providing quality intrapartum care are not optimal.

**Electronic supplementary material:**

The online version of this article (doi:10.1186/s12884-017-1441-2) contains supplementary material, which is available to authorized users.

## Background

Despite failing to reach the millennium development goals (MDGs), much progress has been made in improving the health of mothers and children globally [1]. Maternal mortality ratio (MMR) fell by 44% and under-five mortality rate declined by 53% between 1990 and 2015. Ethiopia registered a more remarkable progress, reducing MMR by 71.8% and meeting the MDG target for reducing under-five mortality by two-thirds [2, 3]. However, the levels of maternal mortality ratio (353 per 100, 000 live births), neonatal mortality rate (28 per 1000 live births) and stillbirth rate (29.7 per 1000 births) remain high, making Ethiopia one of the largest contributors to the global burden of maternal deaths, newborn deaths, and stillbirths, ranking fourth, sixth, and fifth, respectively [4, 5].

In 2015, the United Nations General Assembly adopted the more ambitious sustainable development goals (SDGs), which include targets for ending preventable neonatal deaths and drastically reducing global MMR to less than 70 per 100,000 live births [1]. In line with this global aspiration, the Government of Ethiopia committed to markedly reduce MMR to 199 per 100,000 live births and neonatal mortality (NMR) rate to 10 per 1000 live births by 2020 [6].

Meeting these ambitious global and national goals for maternal and newborn health requires improving the quality of maternal and newborn care. In view of the fact that intrapartum and postpartum periods are the time of greatest risk for the mother, fetus and newborn [7], assuring the quality of care provision during labor, childbirth and immediate postpartum period is of utmost importance. Encouraged by a positive trend in coverage of healthcare services during the MDG period, the Government of Ethiopia has also put unprecedented emphasis on improving quality of care in its current health sector plan [6]. In addition to improving health systems and health outcomes [8], improving quality of care can increase demand for maternal health care [9], which is still a challenge in Ethiopia [10].

Improving quality of care requires measuring it accurately and addressing identified gaps [11]. There is a clear need for more and better research evidence on quality of intrapartum care and quality of maternal health workforce especially from low and middle income countries [12–15]. Most previous studies on quality of care or workforce from Ethiopia and other resource-constrained settings are based on self-report, written test, or simulation with anatomical models [16–23]. In addition, most studies assessed emergency obstetric and newborn care [EmONC] capability but not quality of routine childbirth care [12, 18, 22, 24–28].

The literature on healthcare quality measurement and improvement describe multiple dimensions of healthcare quality. The Donabedian model and its derivatives focus on the structure-process-outcome dimensions as the basis for healthcare quality measurement and improvement, where structure encompasses the physical environment that is conducive to providing quality care, process refers to professional competence of providers and effective communication with clients, and outcome includes mortality, morbidity and patient satisfaction [11, 29–35]. On the other hand, a systematic review of performance measurement and improvement frameworks in health, education and social service sectors identified 16 quality concepts and categorized them under five domains: collaboration, learning and innovation, management perspective, service provision, and outcome [36].

For the purpose of our study, we assessed some elements of quality of intrapartum care described in both models [29, 36]; namely, aspects of structure, process and outcome in the Donabedian framework; and aspects of learning and innovation, management perspective, service provision, and outcome in the cross-sectoral performance improvement framework. Our study also sought to assess quality of care in workplace settings through direct observation. Specifically, we assessed competence of midwives in provision of routine and emergency care during labor, childbirth, and immediate postpartum period including maternal and newborn outcomes. Secondly, we evaluated availability of essential resources for provision of quality labor, delivery, and immediate postpartum care. Thirdly, we assessed availability of opportunities for continuous quality improvement of labor, delivery and immediate postpartum care.

## Methods

### Study design and setting

A cross-sectional study using multiple data collection methods was conducted from 25 January to 14 February 2015 to assess the quality of midwifery care during labor, childbirth and first 6 h of the postpartum period. The study was conducted in government health facilities of the Amhara National Regional State, the second most populous region in Ethiopia, with an estimated population of 20.4 million people [37].

### Study participants

At the time of the study, the Amhara National Regional State had 19 hospitals and 801 health centers owned by the government; and there were 1400 midwives working in these facilities. The inclusion criteria for facilities was having at least two midwives and a caseload of one or more deliveries per day. Accordingly, 19 hospitals and 360 health centers met the inclusion criteria.

Sample size for the number of midwives to be included in the study was estimated to be 150. The sample size was determined (with the formula of n = (Z1-α)^2^ SD^2^ Deff/d^2^) based on the following assumptions: 95% level of confidence, 51.8% mean competence score of midwives with standard deviation (SD) of 15.3% [17], 5% margin of error (d), and design effect (Deff) of 1.2. Since N (number of midwives in facilities with one or more deliveries per day) was 834, a finite population adjustment (n/(1 + n/N)) was applied. Finally, a 10% allowance was considered for anticipated non-response resulting in a sample size of 150.

The study used a two-stage cluster sampling technique, where health facilities were sampled at first stage and midwives sampled at the second stage. Data from the regional health bureau showed, on average, six midwives and two midwives were available in hospitals and health centers, respectively. Assuming four midwives will be recruited from each hospital and two midwives from each health center, 56 health facilities were required to achieve the necessary sample size. Accordingly, all the 19 public hospitals were included in the study while we selected 37 out of the 360 eligible health centers by simple random sampling using computer generated random numbers. (Table 1)

**Table 1:** Sampling of government health facilities and midwives, Amhara Regional State, Ethiopia, 2015

Strata	# of hospitals and health centers	# of facilities with at least one delivery per day	Estimated # of midwives working in eligible facilities	Allocation of midwives by facility type	# of sample facilities
Hospital	19	19	114	76	19
Health center	801	360	720	74	37
Total	820	379	834	150	56

### Data collection

For the purposes of this study, data were collected on the three aspects of the structure-process-outcome model [29] as well as the four aspects of the cross-sectoral performance measurement and improvement framework [36]: competence (which corresponds to the “process” and the “service provision” aspects in the Donabedian and cross-sectoral performance measurement framework, respectively), availability of essential resources for intrapartum care (which falls under the “structure” and the “management perspective” aspects in the Donabedian and cross-sectoral frameworks, respectively), continuous quality improvement practices (which fall under the “learning and innovation” aspect in the cross-sectoral framework), and maternal and newborn outcomes (which are captured in both models).

Data were collected using direct observation of performance, inventory of resources and infrastructure, and structured interview with midwives. Each midwife was observed while providing labor, delivery, and postpartum care to a woman from admission through 6 h after childbirth. If the observation was incomplete, a midwife was observed on the next laboring mother. Performance was assessed for 13 aspects of intrapartum care; namely, rapid initial evaluation, history taking, physical examination, (the modified) partograph use, assisting a woman to have a safe and clean birth, immediate postpartum care, clinical judgment/decision-making, responding to problems, communication skills, infection prevention, organization, efficiency and teamwork, humanistic qualities/professionalism, and overall performance in providing labor, delivery and immediate postpartum care. Proficient midwives performed the rating using a 9-point Likert scale, where 1 to 3 denoted unsatisfactory or incompetent performance, 4 to 6 satisfactory or competent performance, and 7 to 9 outstanding or superior performance. Brief descriptors of typical performance of each aspect were written on the assessment tool to standardize rating. If complications arose during the process of care, assessors evaluated competence of midwives in managing the complications using appropriate checklists adapted from national guidelines (performance rating scales for routine care and checklists for complications management are provided as Additional files 1, 2, 3 and 4).

Data collectors also carried out facility inventory of drugs, medical equipment, supplies, and infrastructure essential to provide care during labor, delivery and postpartum period using an observation checklist. Thirdly, structured interview was conducted with midwives to capture perceived availability of resources and learning and performance improvement opportunities (Interview questionnaire and inventory checklist are annexed as Additional files 1 and 2). The interview took place at a convenient time and place for study participants.

Data were collected by 12 proficient midwives with supervisory support from four members of the research team. Data collectors and supervisors attended training before fieldwork including hands-on practice of observation and performance rating. Actual field pre-testing was also done in health facilities to check and improve reliability of the tools and assessors.

Before beginning data collection, the study team first met the person in charge of each health facility and explained the purpose of the study; presented a letter of approval from the regional health bureau; provided a copy of the study information sheet; and answered questions. Study team members then met all eligible participants at each facility and explained the study and sought written consent from providers and verbal consent from mothers.

### Data analysis

Data were entered into EPI-Data and exported to STATA® IC 12 (STATA Corp. Texas, USA) for analysis. Competence of midwives in providing intrapartum care was determined by calculating average performance scores across the 13 dimensions. The percent of midwives who had unsatisfactory (incompetent), satisfactory (competent) and superior (outstanding) performance for each of the 13 dimensions was calculated. These were summarized by calculating mean percentages for the entire care. Proportion of respondents who managed complications competently was also calculated. Satisfactory and superior performance were interpreted as competent performance. Reliability analysis was performed to assess internal consistency of the items but we could not do inter-rater and intra-rater reliability as the performances were not rated by two independent raters nor twice by the same rater. Proportions were also used to summarize findings of facility inventory and interview on availability of resources and performance improvement opportunities. Percentages of facilities having less than 50%, 50 to 75%, and more than 75% of the resources in each category were computed. Chi-square test was done to identify significant differences between hospitals and health centers. Missing data were excluded from the analysis.

## Results

### Profile of study participants

A total of 150 midwives and 56 government health facilities (37 health centers and 19 hospitals) in which they worked were included in the study, yielding a 100% response rate. However, fewer midwives than planned (57 versus 74) were actually observed from health centers, as some health centers did not have the expected number of midwives or a laboring mother during the facility visit and these were compensated by observing more midwives from hospitals (93 versus 78). Majority of midwives in our study were males, under 25 years of age, with a diploma level training, and with less than 5 years of work experience. Moreover, 57 study participants (38%) were from health centers while 59 (39.3%) were from district or zonal hospitals and 34 (22.7%) from referral hospitals. A significantly higher proportion of study participants from hospitals were bachelor degree holders (*P* < 0.001) (Table 2).

**Table 2:** Socio-demographic characteristics of midwives observed providing labor, delivery and immediate postpartum care, Ethiopia, 2015

Variable	Hospital (*n* = 93)	Health center (*n* = 57)	All facilities (*n* = 150)	*P*-value#
	N (%)	N (%)	N (%)	
Sex				0.941
Male	50 (53.8%)	31 (54.4%)	81 (54%)	
Female	43 (46.2%)	26 (45.6%)	69 (46%)	
Age (*n* = 121)				0.949
20–24 years	35 (49.3%)	26 (52%)	61 (50.4%)	
25–29 years	29 (40.8%)	19 (38%)	48 (39.7%)	
30 years and above	7 (9.9%)	5 (10%)	12 (9.9%)	
Level of education				<0.001
Bachelor	43 (46.2%)	9 (15.8%)	52 (34.7%)	
Diploma	50 (53.8%)	48 (84.2%)	98 (65.3%)	
Experience				0.274
< 24 months	44 (48.3%)	20 (35.1%)	64 (43.2%)	
24–59 months	37 (40.7%)	30 (52.6%)	69 (46.6%	
> =60 months	10 (11%)	7 (12.3%)	17 (11.5%)	

**#**Chi-square test

### Competence in providing labor, delivery, and immediate postpartum care

**Fig. 1:**
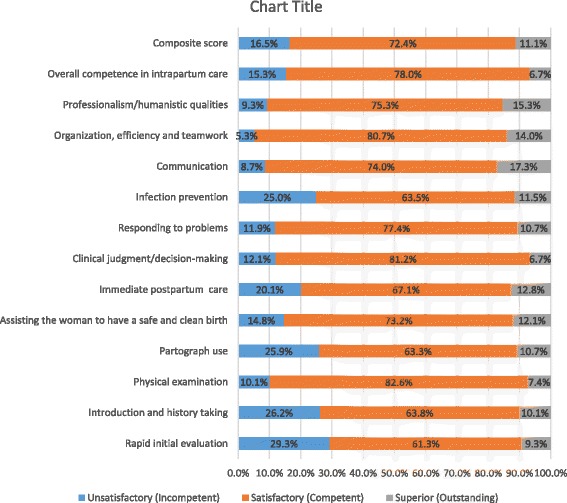
Competence of midwives in providing labor, delivery and immediate postpartum care, Ethiopia, 2015

We estimated proportion of competent midwives based on average performance scores in the 13 domains. Accordingly, 16.5% of midwives were rated incompetent (had unsatisfactory performance), 72.4% were competent (had satisfactory performance), and 11.1% were outstanding (had superior performance). A relatively higher level of unsatisfactory performance (20.1– 29.3%) was observed in rapid initial evaluation, history taking, partograph use, infection prevention, and immediate postpartum care tasks, in descending order. Eleven midwives did not use partograph and were excluded from the analysis on partograph skill. Ten of them decided not to use partograph because the women they attended were in second stage at the time of admission. One provider did not have a partograph in the facility at the time of the study (Fig. 1). We also found that higher percent of midwives working in hospitals were competent than those in health centers; however, the overall difference was not statistically significant [*P* = 0.065] [Table 3]. Reliability [internal consistency] coefficient of the 13 aspects of performance as measured by our tool generated a Cronbach’s Alpha of 0.94.

**Table 3:** Competence difference between hospital and health center midwives in intrapartum care, Ethiopia, 2015

Competencies	No. [%] of competent hospital midwives	No. [%] of competent health center midwives	*P*-value#
Rapid initial evaluation	71 [76.3%]	35 [61.4%]	0.051
Introduction and history taking	76 [82.6%]	34 [59.6%]	***0.002***
Physical examination	88 [94.6%]	46 [82.1%]	***0.014***
Partograph use	68 [80%]	35 [64.8%]	***0.046***
Assist the woman to have a safe and clean birth	79 [85.9%]	48 [84.2%]	0.781
Immediate postpartum care	80 [86.0%]	39 [69.6%]	***0.016***
Clinical judgment/decision-making	82 [88.2%]	49 [87.5%]	0.903
Responding to problems	51 [86.4%]	23 [92.0%]	0.472
Infection prevention	81 [88.0%]	30 [53.6%]	***<0.001***
Communication	86 [92.5%]	51 [89.5%]	0.526
Organization, efficiency and teamwork	90 [96.8%]	52 [91.2%]	0.142
Professionalism/humanistic qualities	85 [91.4%]	51 [89.5%]	0.694
Overall competence in intrapartum care	83 [89.2%]	44 [77.2%]	***0.047***
**Composite score**	79 [87.6%]	41 [76.5%]	0.065

#Chi-square test; statistically significant *p*-values are italicized and bold

### Competence in managing obstetric and newborn complications

**Fig. 2:**
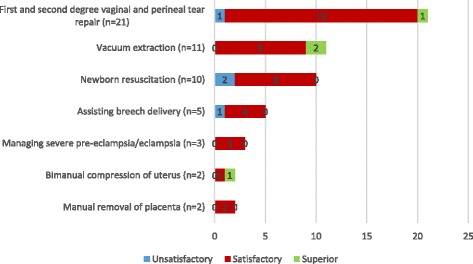
Competence of midwives in managing obstetric and newborn complications, Ethiopia, 2015

A total of 54 obstetric and newborn complications were observed during data collection requiring emergency care. These were first and second degree vaginal and perineal tear, 21 (38.9%), prolonged labor, 11 (20.4%), birth asphyxia, 10 (18.5%), breech presentation, 5 (9.3%), severe pre-eclampsia/eclampsia, 3 (5.5%), retained placenta, 2 (3.7%), and atonic postpartum hemorrhage, 2 (3.7%). We were able to assess performance of 45 midwives (30%) in managing the complications. The most frequently observed emergency care were vaginal and perineal tear repair, 21 (38.9%), vacuum extraction, 11 (20.4%), and neonatal resuscitation, 10 (18.5%). Most midwives, 41 (91%), were judged competent in managing the obstetric and newborn complications. Unsatisfactory performance was observed in newborn resuscitation (2 out of 10), assisting breech delivery (1 out of 5), and tear repair (1 out of 20). Furthermore, three referrals and one newborn death were witnessed during the study **(**Fig. 2 **).**

### Inventory of drugs, medical equipment, and supplies

Inventory of pre-identified resources necessary for labor, delivery and immediate postpartum care found that only 16.3% of facilities had all the essential drugs, 9.6% all the medical equipment, 7.7% all the medical supplies, 6.3% all the infection prevention (IP) materials, and 14.6% all the records and forms. The proportion of facilities with more than 75% of the items in each category was 32.6% for drugs, 73.1% for equipment, 65.4% for supplies, 47.9% for IP materials, and 43.6% for records and forms. A statistically significant difference was observed between hospitals and health centers, favoring the former, in the availability of drugs (*p* = 0.024), medical equipment (*p* = 0.014), IP materials (*p* = 0.002), and records and forms (*p* = 0.034). Facilities about which incomplete or no information on items in a particular domain is provided were excluded from analysis (Table 4).

**Table 4:** Inventory of drugs, medical equipment, medical supplies, infection prevention materials, and records and forms, Ethiopia, 2015

Variables	Hospitals (*n* = 19)	Health centers (*n* = 37)	All facilities (*n* = 56)	*P*-value#
Drugs (8 items)^a^	*n* = 15	*n* = 34	*n* = 49	0.024
< 50%	2 (13.3%)	7 (20.6%)	9 (18.4%)	
50–75%	4 (26.7%)	20 (58.8%)	24 (49.0%)	
> 75%	9 (60%)	7 (20.6%)	16 (32.6%)	
Medical equipment (22 items)^b^	*n* = 16	*n* = 36	*n* = 52	0.014
< 50%	-	2 (5.6%)	2 (3.8%)	
50–75%	-	12 (33.3%)	12 (23.1%)	
> 75%	16 (100%)	22 (61.1%)	38 (73.1%)	
Medical supplies (11 items)^c^	*n* = 18	*n* = 34	*n* = 52	0.39
< 50%	1 (5.6%)	3 (8.8%)	4 (7.7%)	
50–75%	3 (16.6%)	11 (32.4%)	14 (26.9%)	
> 75%	14 (77.8%)	20 (58.8%)	34 (65.4%)	
Infection prevention (IP) materials (16 items)^d^	*n* = 15	*n* = 33	*n* = 38	0.12
< 50%	-	5 (15.1%)	5 (10.4%)	
50–75%	5 (33.3%)	15 (45.5%)	20 (41.7%)	
> 75%	10 (66.7%)	13 (39.4%)	23 (47.9%)	
Records and forms(6 items)^e^	*n* = 19	*n* = 36	*n* = 55	0.034
< 50%	-	-	-	
50–75%	7 (36.8%)	24 (66.7%)	31 (56.4%)	
> 75%	12 (63.2%)	12 (33.3%)	24 (43.6%)	

^a^Drugs include oxytocin, intravenous solutions, magnesium sulfate, calcium gluconate, oxygen gas, adrenaline, lidocaine, and TTC eye ointment

^b^Medical equipment include blood pressure apparatus, thermometer, adult stethoscope, fetoscope, examination table, delivery coach, delivery set, stepping stool, IV stand, watch clock, screen, vaginal speculum, episiotomy kit, suction bulb, ambu bag, infant face mask or suction machine, newborn resuscitation table, radiant warmer, light source, weighing scale, autoclave, and refrigerator

^c^Medical supplies include surgical glove, cord tie, chromic catgut, gauze/cotton, blanket for wrapping newborn, IV cannula, IV sets, needle and syringe, urinary catheter, container for 0.5 chlorine solution, and tape

^d^IP materials include antiseptics/alcohol hand rub, safety box, utility gloves, soap at all sinks, high level disinfectant, alcohol 70%, chlorine solution for decontamination, water, examination glove, single personal use hand towel, tight fitting containers for used linens, tight fitting containers for trash, towels for drying newborns, protective footwear, protective eyewear, and plastic apron

^e^Records and forms include delivery log, partograph, service delivery guidelines, site specific protocols, educational charts and patient documents

#Chi-square test

### Perceptions of the work environment

We assessed reported availability of essential resources for provision of quality labor, delivery, and immediate postpartum care. Availability of records and forms (96.7%) and medical supplies (94%) was reported to be nearly universal. However, only 73.3% respondents said that their facility had basic infrastructure for labor, delivery, and postpartum care (furnished delivery room, neonatal corner, postpartum ward, water, toilet, electricity, and infection prevention facilities). Moreover, 18.7, 14.8, and 23.5% of respondents, respectively, said essential medical equipment, emergency medications, and infection prevention materials were not adequate in their facilities. Although most midwives reported availability of job aids in their health facility, job aids for normal labor and delivery and immediate postpartum care were reported relatively less frequently at 69.8 and 62%, respectively. More hospital than health center midwives reported availability of medical equipment (*P* = 0.021), emergency medications (*p* < 0.001), labor and delivery complications job aids (*P* = 0.001), immediate postpartum care job aids (*P* < 0.001), and newborn problems job aids (*P* < 0.001) (Table 5).

**Table 5:** Perceptions of midwives regarding availability of resources and performance improvement opportunities for labor, delivery and immediate postpartum care, Ethiopia, 2015

Variable	Hospital midwives	Health center midwives	Total	*P*-value***
Job aids on normal labor and delivery (L &D)	70 (75%)	35 (61.4%)	105(69.8%)	0.079
Job aids on managing complications of L&D	83 (89.3%)	39 (68.4%)	122(81.3%)	0.001
Job aids for immediate postpartum care	70 (75.3%)	23 (40.4%)	93(62.0%)	<0.001
Job aid for managing newborn problems	88 (94.6%)	42 (73.7%)	130(86.7%)	<0.001
IP equipment and supplies	76 (80.9%)	40 (70.2%)	116(76.5%)	0.151
Medical equipment	81 (87.1%)	41 (71.9%)	122(81.3%)	0.021
Medical supplies	87 (93.5%)	54 (94.7%)	141 (94%)	0.766
Emergency medications	87 (94.6%)	41 (71.9%)	128(85.2%)	<0.001
Records and forms	88 (94.6%)	57 (100.0)	145(96.7%)	0.075
Basic infrastructure^a^	81 (73.6%)	29 (26.4) %)	110(73.3%)	0. < 0.00
Encounter obstetric complications at least weekly	64 (69.6%)	23 (40.3%)	87(58.4%)	<0.001
Technical update in the last 2 years	65 (69.9%)	46 (80.7%)	111(74.0%)	0.141
Supportive supervision or coaching	48 (51.6%)	44 (77.2%)	92(61.3%)	0.002
Case discussion or seminar^b^	60 (64.5%)	26 (45.6%)	86(57.3%)	0.019
Maternal death review or clinical audit	81 (87.1%)	28 (49.1%)	109(72.7%)	<0.001
Performance-based recognition or reward	23 (24.7%)	19 (33.3%)	42(28.0%)	0.290

^a^Basic infrastructure includes equipped delivery room, neonatal corner, postpartum ward, water and infection prevention facilities, toilet and electricity. ^b^Case presentation, seminar, structured discussion, morning session or grand round. ***Chi-square test

We also assessed perceived availability of learning and performance improvement opportunities. Midwives reported attending an average of two deliveries on daily basis (range from 2 to 3 births per week to 7 births per day); and 62.7% said they encountered obstetric complications or complex cases at least weekly. Majority of respondents reported knowledge and skills update training in the last 2 years (74%), regular supportive supervision visits (61%), structured case discussion about maternal and newborn care (57.3%), and maternal death review or clinical audit in their facility (72.7%). However, fewer percentages of respondents were trained on basic emergency obstetric and newborn care (BEmONC) (31.3%), essential newborn care (ENC) or helping babies breathe (HBB) (26.7%), prevention of mother to child transmission of HIV (PMTCT) (34.7%), and infection prevention (IP) (9.3%). Lack of training was also mentioned as a barrier to give quality labor and delivery services in the open ended question by 25.3% of study participants. Likewise, only 48% said the Ministry of Health (district health office, zonal health department, or regional health bureau) conducted supervisory visits and only 44.7% were visited at least quarterly. Moreover, only 28% reported getting recognition, incentive or reward of any sort for improved performance in labor and delivery services (Table 5).

We found that a higher proportion of respondents from hospitals reported exposure to complicated cases (*P* < 0.001), case discussion (*P* = 0.019), and maternal death review or audit (*P* < 0.001). On the other hand, a higher percent of midwives from health centers than hospitals reported receiving supervision (*p* < 0.002), training, and reward/recognition, although the latter two were not statistically significant (Table 5).

## Discussion

Our findings demonstrate the presence of gaps to provide quality intrapartum care in government health facilities in Amhara Regional State of Ethiopia. There were major deficits in availability of essential physical resources and mechanisms for continuous performance and quality improvement. A significant proportion of midwives were also found incompetent.

Global maternal and newborn health care standards state that competent staff must be available at all times to provide quality care to every woman and every newborn [38]. While it is encouraging that most midwives in our study are competent in providing intrapartum care, the significant proportion of midwives who displayed unsatisfactory performance in routine child birth care (1 in 6), and basic emergency obstetric and newborn care (1 in 11) makes it difficult to guarantee that every mother and every newborn will receive high quality care. It is also noteworthy that more substantial gaps were observed in rapid initial evaluation, history taking, partograph use, infection prevention, assisting normal birth, immediate postpartum care, and newborn resuscitation (Figs. 1 and 2). The World Health Organization guide for essential practice in pregnancy, childbirth, postpartum and newborn care recommends the first five care practices for every woman during childbirth and newborn resuscitation for a baby who is not breathing or is gasping [39]. Systematic review of evidence-based guidelines also recommend partograph use for monitoring labor [40]. While acknowledging health systems weaknesses may limit partograph use and effectiveness, a realist review of the partograph has also suggested that it may improve outcomes in low resource settings [41]. In our study, aside from a quarter of midwives who demonstrated unsatisfactory performance in partograph use, additional ten midwives excused themselves from completing a partograph wrongly thinking there was no need to use a partograph if a woman was in second stage of labor at admission. Our study findings also indicated that midwives working in health centers had larger gaps in their capacity than those from hospitals in almost all domains. However, the difference in the composite score was not statistically significant possibly due to small sample size (Table 3). Although direct comparison is difficult due to differences in methodology, past studies from Ethiopia and other resource-constrained settings have also pointed to shortfalls in competence of midwives to provide intrapartum care [16–22, 24, 42, 43].

While weaknesses in quality of the health workforce are acknowledged to be pervasive, there are also calls for better measurement and improvement of health workforce performance (especially in low and middle in-come countries) to achieve global health development goals [13–15, 44–46]. We believe our use of direct observation to measure performance of midwives in workplace settings responds to the call for better measurement of quality of intrapartum care. The gaps uncovered also warrant strengthening pre-service midwifery education with focus on curriculum review, faculty development, use of simulation methods, and strengthening accreditation and regulation processes, among other things [46].

All midwives have a responsibility to undertake continuing professional development activities [47] and ensuring a high performing midwifery workforce also requires creating a work environment that fosters continuous quality improvement in every facility [38]. Provided effective implementation, in-service training or continuing professional development, supervision and coaching, audit, feedback, and job aids coupled with an enabling environment can improve provider performance [48–54]. However, our results did not show every midwife had sufficient opportunities for in-service training in general and those pertaining to intrapartum care (BEmONC, ENC or HBB, IP, and PMTCT) in particular. While it is surprising that majority of respondents did not receive training on these high priority topics, it demonstrates access to in-service training on intrapartum care remains limited in Ethiopia [18, 42]. One explanation could be that pre-service education systems are producing midwives more rapidly than the capacity of in-service training systems to cope. Another possible explanation is gaps in targeting relevant in-service training to those who need it the most.

Other opportunities for practice-based learning and improvement (like supportive supervision, structured case discussion, clinical audit or maternal death review, job aids, and performance-based reward or incentive) were also found inadequate. Generally speaking, a higher proportion of midwives working in hospitals reported learning and quality improvement opportunities with the exception of supportive supervision visit, which was reported significantly more frequently from health centers. Our findings are consistent with program and study reports that highlighted health systems weaknesses in implementing audit and supportive supervision. Maternal death surveillance and response systems in Ethiopia [6, 55] and globally [56] suffer from inadequate leadership commitment at sub-national level, poor documentation and under-reporting of maternal deaths, fear of blame, and lack of trained staff, among other things. A study of barriers to quality EmONC from Ethiopia has also identified gaps in supervision including, but not limited to, being sporadic, unsupportive, and donor-driven [42]. All these findings indicate the need for strengthening health worker performance and quality improvement strategies in health facilities.

Global standards for improving quality of maternal and newborn care also require health facilities to ensure availability of basic infrastructure and adequate stock of essential equipment, drugs and supplies for intrapartum care [38]. However, the major gaps in availability of essential resources for provision of labor, delivery and immediate postpartum care in our study (Tables 4 and 5) is concerning as it would affect the ability and motivation [57] of midwives to provide quality care to mothers and newborns. A higher proportion of health centers than hospitals had resource gaps. This assumes greater significance when one takes into account the fact that health centers are the primary and most accessible birthing facilities for most women in Ethiopia. In addition to reducing effectiveness of maternal and newborn healthcare, weak infrastructure can undermine the demand to deliver in health centers [58].

Maternal and newborn care surveys from Ethiopia, Tanzania, Uganda, Kenya, Namibia, and Bangladesh have all reported gaps in availability of essential commodities. A basic emergency obstetric and newborn care survey of health centers from Addis Ababa, Ethiopia, found that only 50% had parenteral antibiotics and diazepam; none had magnesium sulfate; and only 90% had a functional vacuum extractor [18]. Inadequate equipment and supplies, and lack of knowledge and skills in performing EmONC were the two main challenges identified in a study of maternity care services in Moshi urban district of northern Tanzania [24]. Another study in Tanzania involving qualitative interviews with nurse-midwives in basic and comprehensive EmONC facilities also revealed that nurse-midwives lacked essential supplies to do their job [25]**.** A health facility-based survey from Karamoja region of Uganda reported lack of equipment and supplies as the most frequent reason for not performing EmONC signal functions and found that 50% of health centers lacked basic equipment for normal delivery and some lacked equipment for neonatal resuscitation as well as consumable supplies and drugs [26]. Emergency obstetric care readiness assessment in rural northwest Bangladesh found that availability of EmONC specific medicines and commodities was 62% in public facilities while coverage for equipment and supplies was 90%. Half of the respondents also mentioned not having essential medicines and commodities in stock as main constraint to EmONC provision [27]. Evaluation of clinical quality of maternal and newborn care in Kenya and Namibia found gaps in essential drugs and commodities including oxytocin, magnesium sulfate, antibiotics, and incubator [28].

Our study findings add to a growing body of literature reporting health system weaknesses to ensure quality of maternal and newborn healthcare. The 2016 Lancet maternal health series has shown access to good quality and evidence-based care remains inadequate especially in low income countries owing to gaps in provider skill and number, facility capability, basic infrastructure for intrapartum care, availability and implementation of evidence-based guidelines, and access to care, among other things [40]. Recent multi-country analyses of health systems bottlenecks in high burden countries have also acknowledged providing quality labor and childbirth care, basic newborn care, and neonatal resuscitation is a challenge, with the most significant weaknesses reported from African countries. Health financing, health workforce, service delivery, and essential commodities related challenges were identified as the major bottlenecks [59, 60]. A systematic review of providers’ perspectives on barriers to quality midwifery care in low and middle income countries have also found professional barrier, which includes, but is not limited to, gaps in education and training, and lack of equipment and supplies, was the most frequently mentioned impediment [61].

### Strengths and limitations

We believe the assessment of quality of care provision during the most critical periods for the mother and the newborn (labor, childbirth, and the immediate postpartum period) makes our study timely and relevant for the global and national maternal and newborn health community. Our attempt to measure the structure (availability of resources for intrapartum care), process (competence of midwives in routine and emergency obstetric and newborn care), and outcome (maternal and newborn morbidity and mortality) dimensions of quality of care as well as strategies for continuous performance and quality improvement is also noteworthy. Moreover, the use of multiple methods including direct observation to measure performance and availability of essential resources lends credibility to our findings. The assessment of quality of both routine childbirth care and emergency care is also important. However, the exclusion of facilities with low volume of delivery services (less than one delivery per day), replacement of some health centers with hospitals (due to challenges with finding expected number of midwives and laboring mothers), and missing data (especially during inventory of commodities) may be considered limitations. Even if we provided brief descriptors of performance in the data collection tool, trained data collectors and conducted pretesting, the subjective judgement involved in performance evaluation can be a source of measurement error but we could not estimate inter-rater or intra-rater reliability. However, internal consistency of the items was found to be very high (Chronbach’s Alpha of 0.94) suggesting the reliability of our results.

## Conclusions

Our study findings indicate the state of the quality of midwifery care during labor, delivery and immediate postpartum period in government health facilities in Amhara Regional State of Ethiopia. Most midwives are competent in routine childbirth care and basic emergency obstetric and newborn care. However, the conditions to provide quality intrapartum care for every woman and newborn cannot be considered optimal. One out of six midwives is not competent in routine childbirth care and one out of 11 midwives is not competent in basic emergency obstetric and newborn care. Many midwives do not have access to sufficient learning and performance improvement opportunities. And most facilities lack essential resources for provision of quality labor, delivery and immediate postpartum care. The gaps seem to be worse in health centers except for supportive supervision and possibly training and performance based recognition. Substantial improvements are needed especially in availability of resources and performance and quality improvement strategies to provide high quality midwifery care during childbirth. Midwifery education should also be strengthened.

## Additional files

Additional file 1: Workplace performance assessment recording tool (Interview questionnaire and direct observation rating scales). (PDF 78 kb)

Additional file 2: Facility inventory checklist. (PDF 113 kb)

Additional file 3: Complications management checklists. (PDF 92 kb)

Additional file 4: Data. (SAV 103 kb)

## Abbreviations

BEmONC: Basic emergency obstetric and newborn care
EmONC: Emergency obstetric and newborn care
ENC: Essential newborn care
HBB: Helping babies breathe
IP: Infection prevention
L&D: Labor and delivery
MMR: Maternal mortality ratio
NMR: Neonatal mortality rate
PMTCT: Prevention of mother to child transmission of HIV
