# Supplementary material for: Quality of Midwife-provided Intrapartum Care in Amhara Regional State, Ethiopia
Source: BMC Pregnancy Childbirth. 2017 Aug 16;17:261. doi: 10.1186/s12884-017-1441-2 (PMC5558781; doi:10.1186/s12884-017-1441-2)
Supplement: Supplementary file 1 — Workplace performance assessment recording tool (Interview questionnaire and direct observation rating scales). (PDF 78 kb) [file 12884_2017_1441_MOESM1_ESM.pdf]

Exempt Determination Date: December 4, 2014 IRB No.: 6118

PERFORMANCE ASSESSMENT OF MIDWIVES IN PROVISION OF CARE DURING LABOR, CHILDBIRTH AND IMMEDIATE POSTPARTUM PERIOD IN TIGRAY AND AMHARA REGIONS, ETHIOPIA

WORKPLACE PERFORMANCE ASSESSMENT RECORDING TOOL: TOOL 1

PART 1: IDENTIFICATION

1. Region name: Amhara…………………1 Tigray……………….2
2. Zone name:__________________
3. Woreda name:____________________
4. Type of health facility: Referral/Regional hospital……………1 Zonal hospital…………2
   District hospital **……………..**3 Health center **………………………….**4
5. Date of interview/ observation (E.C.): day_______ month_______ year 2007

6. Name of observer: _____________________
7. Name of supervisor:_______________________

INSTRUCTION FOR ASSESSORS/DATA COLLECTORS

I. Meet the facility manager and explain the purpose, process and your roles during data collection and obtain permission
II. Along with facility manager and unit coordinator select midwives randomly if there are more than 4 midwives in hospitals and more than 2 midwives in health centers who are on the job at the time your observation
III. Explain the purpose, process and your roles during data collection and obtain permission from both service provider and client
IV. Read the consent paper to the service provider and client to be observed and ask for their permission to be observed (consent paper is attached)
   - If the provider accepts and, signs the consent, ask consent of the client or caretaker
   - If the client or her care taker accepts, continue the data collection
   - If the provider and/or client will not accept, discontinue the observation and proceed to another provider or client if available
V. Take necessary infection prevention measures when observing the provider's performance
VI. Complete **parts 2 & 3** by interviewing the midwife. Record or encircle the responses in the space provided
VII. Complete part 4 by direct observation of performance
   - Observe the provider meticulously while performing tasks
   - For each domain, rate the performance of the midwife as **unsatisfactory**, **satisfactory** or **superior.** Your expert judgment will be required to decide where the performance falls on a scale of 1 to 9, representing spectrum of performance ranging from poorest (1) to best (9).
      i. Put "X" in column **(1, 2, or 3)** if you judge the task performed by the provider is **Unsatisfactory** (**provider does not perform the task** or **attempts to perform the task but is below expectation**)
      ii. Put "X" in column **(4, 5, or 6)** if you judge the task performed by the provider is **Satisfactory** (the **provider performed the task correctly & meets expectations/minimum standards**)
      iii. Put "X" in column **(7, 8, or 9)** if you judge the task performed by the provider is **Superior** (**performance is above expectation and the provider can be considered expert or master, able to teach others**)

PART 2: SOCIO-DEMOGRAPHIC CHARACTERSTICS OF MIDWIVES

No.	Questions	Enter or circle answer	Skip to next questions
1	Observe gender of respondent and circle answer	Male……………….1<br>Female……………2	
2	How old are you? (Age in complete years)	______________Years	
3	What is your highest level of educational qualification (circle one)	Bachelor ………………..………..…1<br>Level IV /Diploma……………………………..……2<br>Masters……………………………….3<br>Other (specify____________)…………9	If answer is 3, Skip to Q6
5	Which type of educational program did you attend?	Generic/Direct ……………………………...1<br>Accelerated……………………………….2<br>Upgrading/Advance Standing………………3	
6	From which institution did you receive your recent professional qualification?	Public university/college……………………1<br>Private university/college……………………2	
7	How long did you serve as midwife? (in years and months	_________years ___________Months	
8	What is your current position /responsibility in the facility and/or Unit? (Multiple responses allowed)	Service provider ………………………..1<br>Unit coordinator ………………………..2<br>Member of facility management ……….3	

PART 3: PERCEIVED AVAILABLITY OF PRACTICE OPPORTUNITIES, RELEVANT TRAINING, PRACTICE-BASED LEARNING, SUPERVISORY AND ORGANIZATIONAL SUPPORT

No.	Questions	Remark	Skip to next questions
**1**	On average, how many deliveries do you attend on daily basis?	___________delivery/deliveries	
**2**	How often do you encounter obstetric complications/complex cases?	Daily………………………………………………………….1<br>Weekly (at least once a week but less than daily)………………2<br>Monthly (at least once in a month but less than weekly)……....3<br>Rarely (less than monthly)……………………………………..4<br>Never…………………………………………………………5	
**3**	Are there standard and up-to-date job aids for assisting normal labor & delivery readily available in your place of work? (Verify through observation)	Yes……………1<br>No……………..2	**If no skip to Q5**

No.	Questions	Remark	Skip to next questions
**4**	If Yes to Q#3, which type/s is/are available?<br><br>(Multiple answers allowed)	Decision trees……………………………………1<br>Flowcharts………………………………………..2<br>Algorithms………………………………………..3<br>Standard operating procedures…………………..…4<br>National service delivery guidelines……………….5<br>Site treatment protocol……………………………..6<br>Others (specify)……………………………………9	
**5**	Are there standard and up-to-date job aids for managing complications of labor & delivery readily available in your place of work? (Verify through observation)	Yes……………1<br>No……………..2	**If no, skip to Q7**
**6**	If Yes to Q#5, which type/s is/are available?<br><br>(Multiple answers allowed)	Decision trees……………………………………1<br>Flowcharts………………………………………..2<br>Algorithms………………………………………..3<br>Standard operating procedures……………….…..4<br>National service delivery guidelines……………….5<br>Site treatment protocol……………………………..6<br>Others (specify)……………………………………9	
**7**	Are there standard and up-to-date job aids for providing immediate postpartum care readily available in your place of work? (Verify through observation)	Yes……………1<br>No……………..2	**If no, skip to Q9**
**8**	If Yes to Q#7, which type/s is/are available?<br><br>(Multiple answers allowed)	Decision trees……………………………………1<br>Flowcharts………………………………………..2<br>Algorithms………………………………………..3<br>Standard operating procedures……………………..4<br>National service delivery guidelines………………..5<br>Site treatment protocol…………………………...…6<br>Others (specify)……………………………………..9	
**9**	Are there standard and up-to-date job aids for managing newborn problems readily available in your place of work? (Verify through observation)	Yes……………1<br>No……………..2	**If no, skip to Q11**
**10**	If Yes to Q#9, which type/s is/are available?<br><br>(Multiple answers allowed)	Decision trees…………………………………...1<br>Flowcharts………………………………………2<br>Algorithms………………………………………3<br>Standard operating procedures……………………4	

<table>
<tr><th>No.</th><th>Questions</th><th>Remark</th><th>Skip to next questions</th></tr>
<tr><td></td><td></td><td>National service delivery guidelines……………….5
Site treatment protocol……………………………..6
Others (specify)………………………………….9</td><td></td></tr>
<tr><td>11</td><td>What resources are adequately available to provide safe and quality labor and delivery services in your place of work?</td><td>
<table>
<tr><th rowspan="2">Type of resources</th><th colspan="2">Circle either yes or no</th></tr>
<tr><th>Yes=1</th><th>No=0</th></tr>
<tr><td>Infection prevention equipment and supplies</td><td>1</td><td>0</td></tr>
<tr><td>Medical equipment (like sphygmomanometer, thermometer, delivery kits, episiotomy sets, vacuum, bag and mask for newborn resuscitation)</td><td>1</td><td>0</td></tr>
<tr><td>Medical supplies (like glove, sutures)</td><td>1</td><td>0</td></tr>
<tr><td>Emergency medications and equipment (like uterotonics, magnesium sulphate, IV solutions, needle, syringe)</td><td>1</td><td>0</td></tr>
<tr><td>Records and forms (Partograph, delivery log or register)</td><td>1</td><td>0</td></tr>
<tr><td>IEC materials for patient education</td><td>1</td><td>0</td></tr>
</table>
</td><td></td></tr>
<tr><td>12</td><td>Do you have suitable infrastructure to provide safe and quality labor and delivery care in your place of work?</td><td>Yes……………1
No……………..2</td><td></td></tr>
<tr><td>13</td><td>Which infrastructure is readily available to provide safe and quality labor and delivery care in your place of work?

(Multiple answers allowed)</td><td>Labor ward (Well illuminated and ventilated with adequate space and number of beds………………………………………………1
Delivery room (Well illuminated and ventilated, clean, adequate space, and partition for privacy)…………………………………2
Toilet in delivery area……………………………………………3
Water ………………..…………………………………………..4
Newborn care unit ………………………………………………5
Midwives' station……………………………………………….6</td><td></td></tr>
<tr><td>14</td><td>Did you receive knowledge & skill update training in the last two years?</td><td>Yes……………1
No……………..2</td><td>If no, skip to Q16</td></tr>
<tr><td>15</td><td>If yes to Q#14, which training did you receive?

(Multiple answers allowed)</td><td>BEmONC ……………………………………………………....1
Essential newborn care, Helping Babies Breath ………………..…2
PMTCT……………………………………………………………3
Infection prevention ……………………………………………...4
Others(specify__________________)……………………………..9</td><td></td></tr>
<tr><td>16</td><td>Do you get regular supportive supervision, mentoring and/or</td><td>Yes……………1
No……………..2</td><td>If no, Skip to Q19</td></tr>
</table>

No.	Questions	Remark	Skip to next questions
	coaching from your supervisor, Woreda Health Office, Zonal Health Department, Regional Health Bureau or health development partners?		
17	If yes to Q16, which organization supervised/ mentored you?<br><br>(Multiple answers allowed)	RHB…………………………………………1<br>ZHD……………………….……………..2<br>WoHO………………………………………3<br>Health development partners…………………4	
18	If yes to Q # 16, how frequent is the supportive supervision?	Monthly……………….…………………………………1<br>Quarterly ……………………………………………...2<br>Biannually …………………………………………….3<br>Yearly …………………………………………………4<br>Other (specify_________________)……………………..9	
19	Do you have case presentation, seminar, structured discussion, morning session, or grand round at your facility regarding maternal and newborn care?	Yes……………1<br>No……………..2	
20	If yes to Q # 18, how frequent is the case discussion, seminar, structured discussion, morning session or grand round?	Monthly…………………………………….....1<br>Quarterly ……………………………………………………2<br>Biannually ……………………………………………………..3<br>Yearly ………………………………………………………4<br>Other (specify_________________)…………………………...9	
21	Is there a clinical audit or review of maternal deaths in your facility?	Yes……………1<br>No……………..2	
22	If yes to Q#21, how frequent is the audit?	Monthly…………..……………………………………..1<br>Quarterly …………………………………………………..2<br>Yearly…… …………………………………………………...3<br>Others (specify_____________)….………………………..…9	

No.	Questions	Remark	Skip to next questions
**23**	Do you get recognition or incentive/reward of any sort for improved performance in labor and delivery services?	Yes…………….1<br>No……………..2	**If no, skip to Q25**
**24**	If yes to Q # 23, what types of motivational schemes are there?<br><br>(Multiple answers allowed)	Feedback (oral or written)……………………………….1<br>Social recognition……………………………………… 2<br>Material recognition……………………………………..3<br>Monetary ………………………………………………. 4<br>Others (specify_____________)….……………………….9	

25. What are the constraints/challenges to providing safe and quality labor and delivery services in your facility?

____

____

____

____

PART 4: DIRECT OBSERVATION. For each domain, put "X" in the appropriate column. Only one response is expected for each measurement on a scale of 1 to 9

**No.**	**Key tasks/domains to be observed and rated**	**Unsatisfactory** (Not performed **OR** Not able to perform the task safely without supervision)			**Satisfactory** (Competently performed the task)			**Superior** (Performed the task at expert level)			**Comments**
		1	2	3	4	5	6	7	8	9	
1	**Perform rapid initial evaluation at first contact (**Ask and look for any danger signs and immediately respond to problems appropriately based on the finding if any)										
2	**Introduction and history taking** (receive and greet the pregnant women, check or ask for the woman's clinical history (personal, obstetric, and medical), current pregnancy & labor history and record										
3	**Perform physical examination** (Vital signs, abdominal exam, pelvic & vaginal examination)										
4	**Use partograph to monitor labor progress** (record vital signs, fetal heart rate, decent, uterine contraction, cervical dilatation, status of membranes, drugs and fluids intake; **AND** use for action/decision)										
5	**Assist the woman to have a safe and clean birth** (Prepare to assist the birth, provide emotional support and reassurance, perform episiotomy only if necessary, assist the mother to deliver, perform active management of the third stage, check for the uterine tone and massage, examine the placenta, membranes and cord, examine the vulva, perineum and vagina for lacerations/tears and repair tears or episiotomy)										

No.	Key tasks/domains to be observed and rated	Unsatisfactory (Not performed **OR** Not able to perform the task safely without supervision)			Satisfactory (Competently performed the task)			Superior (Performed the task at expert level)			Comments
		1	2	3	4	5	6	7	8	9	
6	**Provide immediate postpartum care** (Dry and warm the baby, eye care, cord care, Vitamin K , and monitor the mother's vital signs, uterine tone and bleeding every 15 minutes)										
7	**Clinical judgment/decision-making in providing care** (conduct targeted assessment and collect relevant data; formulate suitable birth management plan; selectively perform appropriate interventions, considering risks and benefits; evaluate the outcome of the intervention, and make appropriate and timely consultation and referral)										
8	**Responding to problems/irregularities if any** (Early detection of problems or irregularities, and respond with appropriate management of problems/complications)<br>**Complete the appropriate checklist for management of complications**										
9	**Infection prevention** (Hand washing, use of personal protective equipment, disposal of sharps & contaminated waste, decontamination of reusable instrument and sterilization)										
10	**Communication** (Establish rapport, obtain consent, explain rationale for examination or intervention, active listening, effective counselling and education, recording and documentation)										

No.	Key tasks/domains to be observed and rated	**Unsatisfactory** (Not performed **OR** Not able to perform the task safely without supervision)			**Satisfactory** (Competently performed the task)			**Superior** (Performed the task at expert level)			**Comments**
		1	2	3	4	5	6	7	8	9	
11	**Organization, efficiency and teamwork (**Prioritizing, timeliness, succinct, and ability to work with other health workers)										
12	**Humanistic qualities/professionalism** (Attending to mother's need for comfort like having a support/companion, can ambulate, has access to food and fluids, and is in a comfortable position both for labor and delivery; respect, compassion, empathy, confidentiality, privacy, modesty)										

13. Outcome of Case Management by the Midwife

13.1 Status at 1st Encounter(Initial contact)	Normal ……………………………………1<br>Abnormal ………………………………….2
13.2 Outcome after management<br><br>(Only one response allowed)	Improved and discharged ………………………………………1<br>Complicated and managed accordingly ……………………………2<br>Complicated and referred/consulted for better care ………………..3<br>Complicated and went for surgery ………………………………...4<br>Mother Died……………………………………………………...5<br>Newborn died…..…………………………………………………...6<br>Don't know….………………………………………………………8<br>Others (specify___________________)……………………………9

14. How do you rate the complexity of the case? Low ………….1 Moderate…………2 High……………3

**15.** Overall, how do you rate clinical competence of the midwife in providing care during labor, childbirth and immediate postpartum period?	Unsatisfactory (Did not provide safe and adequate care)			Satisfactory (Provided safe and adequate care)			Superior (Provided high quality care at expert level)		
	1	2	3	4	5	6	7	8	9

16. What were the strengths of the observed performance?

----

17. What were the weaknesses of the observed performance?

