# Supplementary material for: Quality of Midwife-provided Intrapartum Care in Amhara Regional State, Ethiopia
Source: BMC Pregnancy Childbirth. 2017 Aug 16;17:261. doi: 10.1186/s12884-017-1441-2 (PMC5558781; doi:10.1186/s12884-017-1441-2)
Supplement: Supplementary file 2 — Facility inventory checklist. (PDF 113 kb) [file 12884_2017_1441_MOESM2_ESM.pdf]

PERFORMANCE ASSESSMENT OF MIDWIVES IN PROVISION OF CARE DURING LABOR, CHILDBIRTH AND IMMEDIATE POSTPARTUM PERIOD IN TIGRAY AND AMHARA REGIONS, ETHIOPIA

COMPLICATION MANAGEMENT ASSESSMENT RECORDING TOOL: TOOL 3

PART 1: IDENTIFICATION

1. Region name: Amhara…………………1 Tigray………………2
2. Zone name:__________________
3. Woreda name:____________________
4. Health facility name:________________________
5. Type of health facility: Referral/Regional hospital……………1 Zonal hospital…………2
   District hospital ……………..3 Health center ………………………….4
6. Date of interview/ observation (E.C.): day_______ month_______ year 2007

7. Name of observer: _____________________
8. Name of supervisor:_______________________

Instructions:

- Write the appropriate response in the appropriate column according to how well the task is performed

  If the task is performed correctly put "1" for yes

  if the task is not done or is performed incorrectly put " 0" for no

- Observe performance by type of complication that occurred. Check with tool 1, part 4, task 8 for consistency

Screening question: Did the woman encounter complication/problem during delivery?

Yes……………..1 (Continue observation and fill in the specific problem/irregularity management checklist)

No……………..2 (if no stop)

PART 2: CHECKLISTS FOR COMPLICATION

CHECKLIST 1: BIMANUAL COMPRESSION OF THE UTERUS

	Key Tasks	Circle only one answer	
		**Yes=1**	**No=0**
	**BIMANUAL COMPRESSION OF THE UTERUS**		
1.	Insert one hand into the vagina , form a fist and place the fist into the anterior vaginal fornix and apply pressure against the anterior wall of the uterus	1	0
2.	Place the other hand on the abdomen behind the uterus and Press the abdominal hand deeply into the abdomen and apply pressure against the posterior wall of the uterus	1	0
3.	Maintain compression until bleeding is controlled and the uterus contracts.	1	0
4.	Monitor vaginal bleeding and take the woman's vital signs:(Every 15 minutes for one hour; Then every 30 minutes for two hours)	1	0
5.	Palpate the uterine fundus to ensure that the uterus remains firmly contracted.	1	0

CHECKLIST 2: ASSISTING BREACH DELIVERY

Key Tasks	Circle only one answer	
	Yes=1	No=0
**ASSISTING BREACH DELIVERY**		
1. Ensure that conditions for breech delivery are present	1	0
2. Catheterize the bladder, if necessary	1	0
3. Tell the woman she can bear down with contractions and Let the buttocks deliver until the lower back and then the shoulder blades are seen.	1	0
4. Gently hold the buttocks in one hand, but do not pull.	1	0
5. deliver one leg at a time If the legs do not deliver spontaneously,	1	0
6. Hold the baby by the hips, but do not pull.	1	0
7. Delivery of the Arms	1	0
8. Allow the arm to disengage spontaneously If the arms are felt on the chest	1	0
9. Use Lovset's maneuver If the arms are stretched above the head or folded around the neck,	1	0
10. Deliver the arm that is posterior If the baby's body cannot be turned to deliver the arm that is anterior first	1	0
11. Delivery of the Head	1	0
12. Use the Mauriceau Smellie Veit maneuver to Deliver the head	1	0
13. Check the birth canal for tears and repairs, if necessary. Following delivery	1	0

CHECKLIST 3 : MANAGING PROLAPSED CORD

Key Tasks	Circle only one answer	
	**Yes=1**	**No=0**
**MANAGING PROLAPSED CORD**		
1. Give oxygen 4 -6 L per minute by mask or through nasal catheter	1	0
2. Place one gloved hand into the vagina and push the presenting part upward.	1	0
3. Hold the presenting part firmly out of the pelvic brim with the abdominal hand until woman has been prepared for cesarean section.	1	0

CHECKLLLIST 4: MANAGING SEVERE PRE-ECLAMPSIA/ECLAMPSIA

Key Tasks	Circle only one answer	
	**Yes=1**	**No=0**
**MANAGING SEVERE PRE-ECLAMPSIA/ECLAMPSIA**		
**Giving Magnesium Sulphate**	1	0
1. Give 4gm of magnesium sulphate (20ml of 20% solution) IV slowly over 20 minutes	1	0
2. Together with IV loading dose, give 10 gm of Magnesium sulphate. Give 5gm (10 ml of 50% solution) IM deep in upper outer quadrant of each buttock with 1ml of 2% lidocaine solution in same syringe	1	0
3. If IV medication is not possible, give the IM dose only as loading dose	1	0
4. If convulsion recurs after 15 minutes, give additional 2 gm of magnesium sulphate(10 ml of 20% solution)IV over 20 mins	1	0
5. If convusion recurs still, give diazepam	1	0
6. If the woman is late in labor or referral delayed, continue treatment with 5gm of 50% magnesium sulphate IM in 1ml of 2% lidocaine solution every four hours in alternate buttock until 24 hours after birth or last convulsion	1	0
7. Monitor toxicity regular using urine output(>100ml/4hrs, RR (>16/min and Knee jerk present)	1	0
8. Don't give next dose if signs are beyond the above levels and knee jerk present	1	0
9. Prepare calcium gluconate antidote for toxicity	1	0
**Giving Diazepam (if toxicity or absence of magnesium sulphate**	1	0
10. Give diazepam 10 mg IV or rectally slowly over 2 minutes	1	0
11. If convulsion recurs, repeat 10 mg	1	0
12. Give maintenance dose of diazepam slowly (40 mg in 500 saline or R/L over 6 – 8 hour	1	0
13. Stop maintenance dose and ventilate if RR< 16/min	1	0
**Giving Antihypertensives**	1	0
14. If diastolic blood pressure > 110mmHg, give hydralazine5mg IV/IM slowly (3 -4min)	1	0
15. If the diastolic BP remains > 90mmHg, repeat the dose at 30 minutes intervals until it becomes around 90 mmHg	1	0
16. Record findings and drugs taken	1	0

CHECKLIST 5: MANUAL REMOVAL OF PLACENTA

Key Tasks	Circle only one answer	
	Yes=1	No=0
**MANUAL REMOVAL OF PLACENTA**		
1. Give anesthesia (pethidine and diazepam IV slowly or ketamine).	1	0
2. Give a single dose of prophylactic antibiotics:<br>3. Ampicillin 2 g IV PLUS metronidazole 500 mg IV OR Ceftriaxone 1gm stat	1	0
4. Hold the umbilical cord with a clamp.	1	0
5. Pull the cord gently until it is parallel to the floor.	1	0
6. Insert the other hand into the vagina along the cord and up into the uterus.	1	0
7. Detach the placenta from the implantation site , hold the placenta and slowly withdraw the hand from the uterus, bringing the placenta with it;	1	0
8. With the other hand, continue to provide counter-traction to the fundus by pushing it in the opposite direction of the hand that is being withdrawn	1	0
9. Give oxytocin 20 units in 1 L IV fluid (normal saline or Ringer's lactate) at 60 drops/minute	1	0
10. Massage the fundus to encourage a tonic uterine contraction.	1	0
11. Give Ergometrine 0.2 mg IM, or give prostaglandins (If there is continued heavy bleeding)	1	0
12. Examine the uterine surface of the placenta to ensure that it is complete:<br>13. • If any placental lobe or tissue is missing, explore uterine cavity to remove it.	1	0
14. Monitor vaginal bleeding and take the woman's vital signs every 30 minutes for the next six hours.	1	0
15. Palpate the uterine fundus to ensure the uterus remains contracted	1	0

CHECKLIST 6: NEWBORN RESUSCITATION

Key Tasks	Circle only one answer	
	Yes=1	No=0
**NEWBORN RESUSCITATION**		
1. Place the baby on her/his back on a clean, warm surface and keep covered except for the face and chest.	1	0
2. Position the head in a slightly extended position to open the airway.	1	0
3. Clear the airway by suctioning the mouth first and then the nose.	1	0
4. Place the mask on the baby's face so that it covers the chin, mouth, and nose.	1	0
5. Squeeze the bag with two fingers only or with the whole hand, depending on the size of the bag and ventilate for 1 min (at a rate of 40 breaths per minute) and observe the chest for an easy rise and fall	1	0
6. Check the seal by ventilating two or three times and observing the rise of the chest	1	0
7. Ventilate at a rate of 40 breaths per minute; If the baby's chest is rising	1	0
8. Check the position of the head , Reposition the mask on the baby's face , Squeeze the bag with the whole hand, Repeat suction of mouth and nose …..If the baby's chest is not rising	1	0
9. Stop ventilating and observe the baby's respiratory rate for five minutes If the baby starts crying after and keep the baby skin-to-skin with the mother	1	0
10. Continue ventilation with oxygen, organize transfer and refer baby to a tertiary care center, if possible; If the baby is not breathing regularly after 20 minutes of ventilation,	1	0
11. Stop ventilating, and provide emotional support to mother and family -If there is no gasping or breathing at all after 20 minutes of ventilation	1	0

CHECKLIST 7: REPAIR OF 1ST AND 2ND DEGREE VAGINAL AND PERINEAL TEARS

Key Tasks	Circle only one answer	
	Yes=1	No=0
**REPAIR OF 1ST AND 2ND DEGREE VAGINAL AND PERINEAL TEARS**		
1. Cleanse perineum with antiseptic solution.	1	0
2. Inject lidocaine (10 ml of 0.5% lidocaine) into vaginal mucosa, beneath skin of perineum and deeply into perineal muscle	1	0
3. Wait two minutes and then pinch the area with forceps, (If the woman feels the pinch, wait two more minutes and then retest)	1	0
4. Use a continuous suture from apex downward to level of vaginal opening	1	0
5. Use 2-0 interrupted sutures to repair perineal muscle, working from top of perineal tear downward	1	0
6. Use interrupted or subcuticular sutures to bring together skin edges.	1	0
7. Wash perineal area with antiseptic, pat dry, and place a sterile sanitary pad over the vulva and perineum	1	0

CHECKLIST 8: VACUUM EXTRACTION

Key Tasks	Circle only one answer	
	Yes=1	No=0
**VACUUM EXTRACTION**		
1. Clean the vulva with antiseptic solution.	1	0
2. Catheterize the bladder, if necessary.	1	0
3. Check all connections on the vacuum extractor and test the vacuum.	1	0
4. Assess the position of the fetal head and identify the posterior fontanelle.	1	0
5. Apply the largest cup that will fit.	1	0
6. Perform episiotomy if necessary	1	0
7. Check the application and ensures that there is no maternal soft tissue within the rim of the cup.	1	0
8. Have assistant create a vacuum of negative pressure and check the application of the cup.	1	0
9. Increase the vacuum to the maximum and then apply traction. Correct the tilt or deflexion of the head.	1	0
10. With each contraction, apply traction in a line perpendicular to the plane of the cup rim and assess potential slippage and descent of the vertex.	1	0
11. Between each contraction, have assistant check fetal heart rate and application of the cup.	1	0
12. Continue the "guiding" pulls for a maximum of 30 minutes. Release the vacuum when the head has been delivered.	1	0
13. Check the birth canal for tears following delivery, and repair if necessary. Repair the episiotomy, if one was performed.	1	0
