# Supplementary material for: Quality of Midwife-provided Intrapartum Care in Amhara Regional State, Ethiopia
Source: BMC Pregnancy Childbirth. 2017 Aug 16;17:261. doi: 10.1186/s12884-017-1441-2 (PMC5558781; doi:10.1186/s12884-017-1441-2)
Supplement: Supplementary file 3 — Complications management checklists. (PDF 92 kb) [file 12884_2017_1441_MOESM3_ESM.pdf]

Exempt Determination Date: December 4, 2014 IRB No.: 6118

MIDWIVES COMPETENCY ASSESSMENT AT HEALTH FACILITY IN TIGRAY AND AMHARA REGIONS

INVENTORY OF ESSENTIAL MEDICAL EQUIPMENT, SUPPLIES AND DRUGS FOR LABOR, DELIVERY, IMMEDIATE POSTPARTUM AND NEWBORN SERVICES RECORDING TOOL: TOOL 2

PART 1: IDENTIFICATION

1. Region name: Amhara………………..1 Tigray……………….2
2. Zone name:__________________
3. Woreda name:____________________
4. Health facility name:________________________
5. Type of health facility: Referral hospital…………..1 Regional/zonal hospital…………2
   District hospital **……………..**3 Health center **……………………….**4
6. Date of / observation (EC): day_______ month_______ year 2007
7. Name of observer: _____________________
8. Name of supervisor:_______________________

Instructions to Assessor:

- Complete this form through direct observation and interview with appropriate staff for the inventory of essential medical equipment, drugs and supplies that affect the performance of midwives in labor and delivery wards.
- Check for the availability, adequacy and functionality each item
- Record "YES=1" if an item is available, **adequate and/or functional** as expected in the corresponding column. Otherwise, you record "NO=0".
- Please write your comments and other relevant information collected during the assessment.

PART 2: LIST OF EQUIPMENT, SUPPLIES AND DRUGS

Equipment/supplies/drugs	Availability (Circle either yes or no)		Adequacy (Circle either yes or no)		Functionality (Circle either yes or no)		Remark
	Yes=1	No=0	Yes=1	No=0	Yes=1	No=0	
Adult stethoscope	1	0	1	0	1	0	

Equipment/supplies/drugs	Availability (Circle either yes or no)		Adequacy (Circle either yes or no)		Functionality (Circle either yes or no)		Remark
	Yes=1	No=0	Yes=1	No=0	Yes=1	No=0	
Clinical thermometer	1	0	1	0	1	0	
Blood pressure apparatus	1	0	1	0	1	0	
Fethoscope (pinard/ electronic doppler)	1	0	1	0	1	0	
Examination table or bed with washable plastic cover	1	0	1	0	1	0	
Delivery table	1	0	1	0	1	0	
Step stool	1	0	1	0	1	0	
Light source (lamp or hand torch)	1	0	1	0	1	0	
IV stand	1	0	1	0	1	0	
Screens	1	0	1	0	1	0	
Delivery set	1	0	1	0	1	0	
Cord ties or clamp	1	0	1	0	1	0	
Chromic, cut gut or suturing material	1	0	1	0	1	0	
Gauze/cotton wool	1	0	1	0	1	0	
Amniotic rapture kit	1	0	1	0	1	0	
Episiotomy/tear repair kit	1	0	1	0	1	0	
Oxygen tubing	1	0	1	0	1	0	
Radiant warmer/heating lamp	1	0	1	0	1	0	
Refrigerator or cold box (for storage of drugs and vaccines)	1	0	1	0	1	0	
weighing Scales (newborn & Adult)	1	0	1	0	1	0	
Vaginal specula of various sizes (small, medium & large)	1	0	1	0	1	0	
Watch or clock with second hand that can be seen easily	1	0	1	0	1	0	
Blanket for wrapping the newborn	1	0	1	0	1	0	
Blankets and/or towels for drying the newborn (indicate number of each)	1	0	1	0	1	0	
Suction bulb or suction machine :	1	0	1	0	1	0	
Newborn resuscitation table	1	0	1	0	1	0	
Ambu bag	1	0	1	0	1	0	
Infant face mask(size 0,1, 2)	1	0	1	0	1	0	
Lidocaine 1% without epinephrine/lidocaine 2% + sterile water or normal saline	1	0	1	0	1	0	
Adrenaline	1	0	1	0	1	0	
Calcium gluconate	1	0	1	0	1	0	
Magnesium sulfate	1	0	1	0	1	0	

Equipment/supplies/drugs	Availability (Circle either yes or no)		Adequacy (Circle either yes or no)		Functionality (Circle either yes or no)		Remark
	Yes=1	No=0	Yes=1	No=0	Yes=1	No=0	
Oxytocin 10IU	1	0	1	0	1	0	
TTC / Silver nitrate eye ointment	1	0	1	0	1	0	
IV solutions: Ringer's lactate, normal saline, glucose	1	0	1	0	1	0	
IV administration sets	1	0	1	0	1	0	
Large bore needles or cannula (16 gauge)	1	0	1	0	1	0	
Needles, syringes different size (3ml, 5ml, 10ml)	1	0	1	0	1	0	
Scissors	1	0	1	0	1	0	
Tape	1	0	1	0	1	0	
Examination gloves	1	0	1	0	1	0	
High-level disinfected or sterile surgical gloves	1	0	1	0	1	0	
Urinary catheters (single use/reuse); (Straight Foley with drainage bag)	1	0	1	0	1	0	
Autoclave/Steam sterilizer	1	0	1	0	1	0	
Antiseptic/Alcohol hand rub	1	0	1	0	1	0	
Water source with wash basins with faucet	1	0	1	0	1	0	
Container for 0.5% chlorine solution for decontamination	1	0	1	0	1	0	
Container for soap solution	1	0	1	0	1	0	
Container for rinsing instruments	1	0	1	0	1	0	
Containers with tight-fitting lids or plastic bags for trash	1	0	1	0	1	0	
Containers with tight-fitting lids or plastic bags for used linen collection	1	0	1	0	1	0	
Alcohol/Betadine/Savlon (circle all that apply)	1	0	1	0	1	0	
Chlorine for making decontamination solution	1	0	1	0	1	0	
Plastic aprons/cover gowns	1	0	1	0	1	0	
Protective eyewear (face shields, goggles)	1	0	1	0	1	0	
Protective footwear (boots/shoe covers)	1	0	1	0	1	0	
Puncture-proof container for sharps disposal	1	0	1	0	1	0	
Single (personal) use hand towel	1	0	1	0	1	0	
Soap at all sinks	1	0	1	0	1	0	
Utility or heavy-duty household gloves for cleaning	1	0	1	0	1	0	

Equipment/supplies/drugs	Availability (Circle either yes or no)		Adequacy (Circle either yes or no)		Functionality (Circle either yes or no)		Remark
	Yes=1	No=0	Yes=1	No=0	Yes=1	No=0	
Appropriate documentation sheets for narrative notes/ Client card	1	0	1	0	1	0	
Delivery log or register	1	0	1	0	1	0	
Partograph forms	1	0	1	0	1	0	
National service delivery guidelines & standards	1	0	1	0	1	0	
Site specific management protocol	1	0	1	0	1	0	
Educational charts, posters and flow charts and cuecards	1	0	1	0	1	0	
